# Supplementary material for: Evaluating the legacy of drought exposure on root and rhizosphere bacterial microbiomes over two plant generations
Source: Microbiol Spectr. 2026 Mar 20;14(4):e03019-25. doi: 10.1128/spectrum.03019-25 (PMC13055371; doi:10.1128/spectrum.03019-25)
Supplement: Supplemental material — Fig. S1; Tables S1 to S14. [file spectrum.03019-25-s0001.pdf]

Supplemental Material

Table S1. Parameters used in 16S rRNA amplicon sequence processing

Dataset	Dada2 trim parameters	Taxonomy assignment	Decontam parameters	Rarefaction
**Michigan, USA Roots 16S**	--p-trunc-len-f 114 \ --p-trunc-len-r 161 \	silva-138-99-515-806-nb-classifier.qza	Threshold 0.1	20,976
**Michigan, USA Rhizosphere 16S**	--p-trunc-len-f 103 \ --p-trunc-len-r 172 \	silva-138-99-515-806-nb-classifier.qza	Threshold 0.1	20,976
**Pays de la Loire, France Root and Rhizosphere 16S - Generation 1**	--p-trunc-len-f 200 \ --p-trunc-len-r 180- \	Silva 132 99%	Threshold 0.2	2,500
**Pays de la Loire, France Root and Rhizosphere 16S - Generation 2**	--p-trunc-len-f 200 \ --p-trunc-len-r 130- \	Silva 132 99%	Threshold 0.2	2,500

Figure S1. Rarefaction curves of number of amplicon sequence variants (ASVs) "Species Richness" by number of reads ("Sample Size") for each microbiome profile generated by 16S rRNA amplicon sequencing. Rhizosphere samples are provided in pink and root in blue. MSU shows samples from Michigan State University, Michigan, USA, and INRAE shows samples from Pays de la Loire, France. Vertical lines show the subsampling depth applied for alpha and beta diversity analyses (20,976 reads per sample for Michigan, and 2,500 reads per sample for Pays de la Loire). Note differences in both y- and x-axis ranges across the two panels.

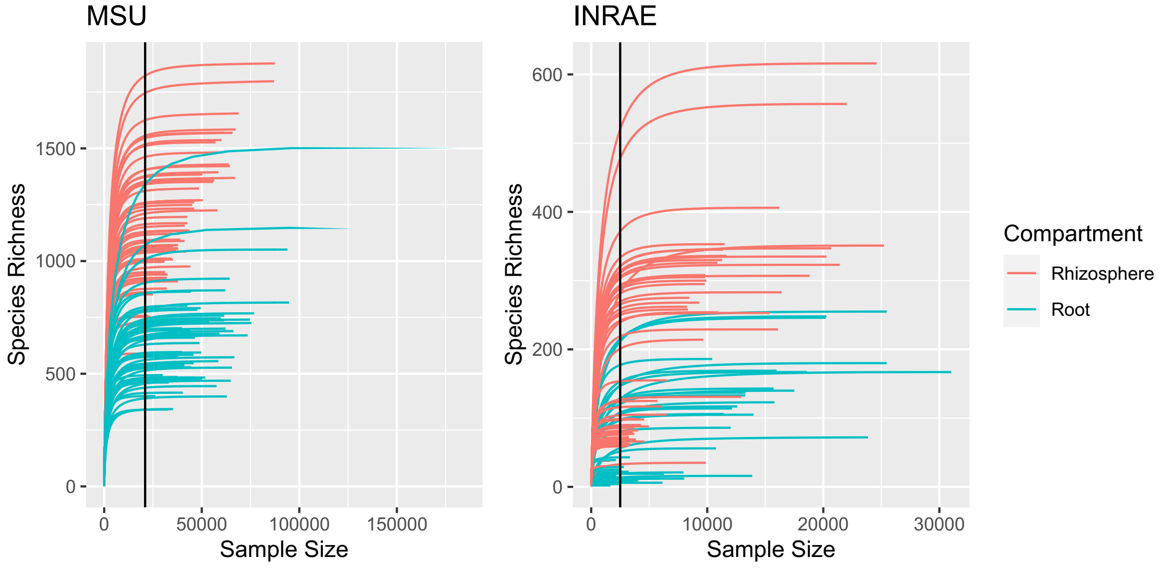

Table S2. Effects of drought treatment, plant genotype, and their interaction on generation 1 (G1) plant phenotypes (pod count, seed count, above-ground and root dry mass, photosynthetic rate, and stomatal conductance) were assessed by two-way ANOVA (Pays de la Loire, France) and Welch's two-sample t-test (Michigan, USA) (df = degrees of freedom, F = F-statistic, t = t-statistic, P = P-value, values P<0.05 are indicated in bold)

Two-way ANOVA	Pays de la Loire (France)			Welch's Two Sample t-test	Michigan (USA)		
	df	F	P		df	t	P
**Pod count**				**Pod count**			
Genotype	1	355.618	**2.36e-12**	*G1 drought*	5.96	0	1
G1 drought	1	58.066	**1.04e-06**				
Genotype x G1 drought	1	0.776	0.391				
**Seed count**				**Seed count**			
Genotype	1	100.292	**2.69e-08**	*G1 drought*	7.6	0.071	0.946
G1 drought	1	0.082	0.778				
Genotype x G1 drought	1	0.002	0.968				
**Above-ground mass**				**Above-ground mass**			
Genotype	1	3.304	0.0891	*G1 drought*	7.96	2.497	**0.037**
G1 drought	1	5.058	**0.04**				
Genotype x G1 drought	1	0.459	0.5084				
**Root mass**				**Root mass**			
Genotype	1	7.648	**0.0138**	*G1 drought*	6.37	1.189	0.277
G1 drought	1	2.377	0.1426				
Genotype x G1 drought	1	0.017	0.8985				
				**Photosynthetic rate**			
				G1 drought	5.71	3.283	**0.018**
				**Stomatal conductance**			
				G1 drought	6.16	2.816	**0.029**

Table S3. Effects of drought treatment on generation 1 (G1) plant phenotypes (pod count, seed count, above-ground and root dry mass) within each genotype in Pays de la Loire, France were assessed by Welch's two-sample t-test (df = degrees of freedom, t = t-statistic, P = P-value, values P<0.05 are indicated in bold)

Welch's two-sample t-test	Pays de la Loire (France)					
	Flavert			Red Hawk		
	df	t	P	df	t	P
**Pod count**						
G1 drought	7.5955	7.0602	**0.0001363**	6.6301	4.264	**0.00422**
**Seed count**						
G1 drought	7.6164	0.13001	0.8999	7.2645	0.49237	0.637
**Above-ground mass**						
G1 drought	4.1567	1.7858	0.146	4.4113	1.4781	0.207
**Root mass**						
G1 drought	5.8955	0.94479	0.3819	7.9852	1.5139	0.1686

Table S4. Effects of drought treatment (G1 drought = parent drought treatment, G2 drought = current drought treatment), plant genotype, and their interactions on generation 2 (G2) plant phenotypes (pod count, seed count, shoot and root dry mass, photosynthetic rate, and stomatal conductance) were assessed by three-way ANOVA (Pays de la Loire, France) and two-way ANOVA (Michigan, US) (df = degrees of freedom, F = F-statistic, P = P-value, values P<0.05 are indicated in bold)

**Three-way ANOVA**	**Pays de la Loire (France)**			**Two-way ANOVA**	**Michigan (USA)**		
	**df**	**F**	**P**		**df**	**F**	**P**
**Pod count**				**Pod count**			
Genotype	1	419.767	**< 2e-16**	*G1 drought*	1	2.038	0.161
G1 drought	1	0.419	0.522248	*G2 drought*	1	0.233	0.632
G2 drought	1	111.674	**5.77e-12**	*G1 drought x G2 drought*	1	0.442	0.510
Genotype x G1 drought	1	0.419	0.522248				
Genotype x G2 drought	1	16.791	**0.000266**				
G1 drought x G2 drought	1	0.047	0.830617				
Genotype x G1 drought x G2 drought	1	1.163	0.288949				
**Seed count**				**Seed count**			
Genotype	1	921.891	**< 2e-16**	*G1 drought*	1	0.013	0.909
G1 drought	1	2.189	0.148815	*G2 drought*	1	0.791	0.379
G2 drought	1	169.851	**2.42e-14**	*G1 drought x G2 drought*	1	1.362	0.250
Genotype x G1 drought	1	0.045	0.833961				
Genotype x G2 drought	1	17.275	**0.000225**				
G1 drought x G2 drought	1	0.124	0.726971				
Genotype x G1 drought x G2 drought	1	2.625	0.114986				
**Above-ground mass**				**Above-ground mass**			
Genotype	1	3.061	0.0898	*G1 drought*	1	0.003	0.958
G1 drought	1	8.953	**0.0053**	*G2 drought*	1	0.180	0.674
G2 drought	1	368.450	**<2e-16**	*G1 drought x G2 drought*	1	1.221	0.275
Genotype x G1 drought	1	3.870	0.0579				
Genotype x G2 drought	1	1.369	0.2507				
G1 drought x G2 drought	1	0.001	0.9754				
Genotype x G1 drought x G2 drought	1	0.227	0.6371				
**Root mass**				**Root mass**			
Genotype	1	8.192	**0.007361**	*G1 drought*	1	0.390	0.536
G1 drought	1	3.585	0.067364	*G2 drought*	1	0.070	0.793
G2 drought	1	14.801	**0.000537**	*G1 drought x G2 drought*	1	0.027	0.869
Genotype x G1 drought	1	2.416	0.129973				
Genotype x G2 drought	1	13.645	**0.000820**				
G1 drought x G2 drought	1	0.001	0.974010				
Genotype x G1 drought x G2 drought	1	0.692	0.411673				
				**Photosynthetic rate**			

G1 drought	1	0.009	0.926087
G2 drought	1	18.425	**0.000642**
G1 drought x G2 drought	1	0.211	0.652636
**Stomatal Conductance**			
G1 drought	1	0.177	0.68
G2 drought	1	46.803	**5.59e-06**
G1 drought x G2 drought	1	0.154	0.70

Table S5. Effects of G1 drought (parent drought treatment), G2 drought (current drought treatment), and their interaction on generation 2 (G2) plant phenotypes (pod count, seed count, above-ground and root dry mass) within each genotype in Pays de la Loire, France were assessed by two-way ANOVA (df = degrees of freedom, F = F-statistic, P = P-value, values P<0.05 are indicated in bold)

Two-way ANOVA	Pays de la Loire (France)					
	Flavert			Red Hawk		
	df	F	P	df	F	P
**Pod count**						
G1 drought	1	0	1	1	1.125	0.305
G2 drought	1	85.630	**7.99e-08**	1	28.125	**7.14e-05**
G1 drought x G2 drought	1	0.296	0.594	1	1.125	0.305
**Seed count**						
G1 drought	1	0.83	0.376	1	2.893	0.108
G2 drought	1	85.79	**7.89e-08**	1	141.750	**2.31e-09**
G1 drought x G2 drought	1	1.13	0.304	1	2.893	0.108
**Above-ground mass**						
G1 drought	1	0.410	0.531	1	17.106	**0.000776**
G2 drought	1	161.863	**8.78e-10**	1	225.981	**7.4e-11**
G1 drought x G2 drought	1	0.077	0.784	1	0.179	0.677861
**Root mass**						
G1 drought	1	8.434	**0.0104**	1	0.044	0.835701
G2 drought	1	0.017	0.8989	1	21.952	0.000248
G1 drought x G2 drought	1	0.453	0.5105	1	0.289	0.598514

Table S6. Effects of drought treatment, plant genotype, and their interaction on root and rhizosphere alpha diversity (richness) of generation 1 (G1) plant were assessed by two-way ANOVA (Pays de la Loire, France) and Welch's two-sample t-test (Michigan, USA) (df = degrees of freedom, F = F-statistic, t = t-statistic, P = P-value, values P<0.05 are indicated in bold)

Two-way ANOVA	Pays de la Loire (France)			Welch's Two Sample t-test	Michigan (USA)		
	df	F	P		df	t	P
**Root**				**Root**			
Genotype	1	7.651	**0.0138**	*G1 drought*	6.87	0.055	0.958
G1 drought	1	3.303	0.0879				
Genotype x G1 drought	1	3.534	0.0785				
**Rhizosphere**				**Rhizosphere**			
Genotype	1	3.395	0.084	*G1 drought*	4.067	0.792	0.472
G1 drought	1	0.271	0.61				
Genotype x G1 drought	1	3.267	0.0895				

Table S7. Effects of drought treatment (G1 drought = parent drought treatment, G2 drought = current drought treatment), plant genotype, and their interactions on root and rhizosphere alpha diversity (richness) of generation 2 (G2) plant were assessed by three-way ANOVA (Pays de la Loire, France) and two-way ANOVA (Michigan, USA) (df = degrees of freedom, F = F-statistic, P = P-value, values P<0.05 are indicated in bold)

Three-way ANOVA	Pays de la Loire (France)			Two-way ANOVA	Michigan (USA)		
	df	F	P		df	F	P
**Root**				**Root**			
Genotype	1	2.490	0.1267	*G1 drought*	1	0.151	0.699
G1 drought	1	0.228	0.6372	*G2 drought*	1	2.422	0.127
G2 drought	1	7.023	**0.0135**	*G1 drought x G2 drought*	1	1.452	0.235
Genotype x G1 drought	1	0.650	0.4275				
Genotype x G2 drought	1	1.556	0.2234				
G1 drought x G2 drought	1	1.188	0.2857				
Genotype x G1 drought x G2 drought	1	0.034	0.8557				
**Rhizosphere**				**Rhizosphere**			
Genotype	1	4.983	**0.03378**	*G1 drought*	1	0.685	0.412
G1 drought	1	9.132	**0.00532**	*G2 drought*	1	0.729	0.397
G2 drought	1	0.266	0.61032	*G1 drought x G2 drought*	1	4.145	**0.047**
Genotype x G1 drought	1	1.336	0.25748				
Genotype x G2 drought	1	0.574	0.45490				
G1 drought x G2 drought	1	1.136	0.29555				
Genotype x G1 drought x G2 drought	1	2.506	0.12463				

Table S8. Effects of G1 drought (parent drought treatment), G2 drought (current drought treatment), and their interaction on generation 2 (G2) on root and rhizosphere alpha diversity (richness) of generation 2 (G2) plant within each genotype in Pays de la Loire, France were assessed by two-way ANOVA (df = degrees of freedom, F = F-statistic, P = P-value, values P<0.05 are indicated in bold)

Two-way ANOVA	Pays de la Loire (France)					
	Flavert			Red Hawk		
	df	F	P	df	F	P
**Root**						
G1 drought	1	1.470	0.245	1	0.001	0.976
G2 drought	1	0.456	0.511	1	8.734	**0.012**
G1 drought x G2 drought	1	0.300	0.593	1	1.158	0.303
**Rhizosphere**						
G1 drought	1	25.631	**0.000173**	1	1.148	0.302
G2 drought	1	1.651	0.219691	1	0.016	0.901
G1 drought x G2 drought	1	2.583	0.130349	1	1.217	0.289

Table S9. Effects of drought treatment, plant genotype, and their interaction on beta diversity (Bray-Curtis distance matrix) of generation 1 (G1) plant were assessed by PERMANOVA (df = degrees of freedom, R^2 = coefficient of determination, F = F-statistic, P = P-value, number of permutations = 9999, values P<0.05 are indicated in bold)

**PERMANOVA**	**Pays de la Loire (France)**				**PERMANOVA**	**Michigan (USA)**			
	**df**	**R^2**	**F**	**P**		**df**	**R^2**	**F**	**P**
**Root**					**Root**				
Genotype	1	0.48604	17.0862	**0.0001**	*G1 drought*	1	0.15159	1.4294	0.1398
G1 drought	1	0.03054	1.0735	0.3092					
Genotype x G1 drought	1	0.02829	0.9944	0.3329					
**Rhizosphere**					**Rhizosphere**				
Genotype	1	0.06119	1.2232	0.2185	*G1 drought*	1	0.10282	0.9168	0.4316
G1 drought	1	0.08592	1.7175	**0.0241**					
Genotype x G1 drought	1	0.05246	1.0487	0.2943					

Table S10. Effects of drought treatment and their interaction on beta diversity (Bray-Curtis distance matrix) of generation 1 (G1) plant within each genotype in Pays de la Loire, France were assessed by PERMANOVA (df = degrees of freedom, R^2 = coefficient of determination, F = F-statistic, P = P-value, number of permutations = 9999, values P<0.05 are indicated in bold)

PERMANOVA	Pays de la Loire (France)							
	Flavert				Red Hawk			
	df	R^2	F	P	df	R^2	F	P
**Root**								
G1 drought	1	0.11572	1.0469	0.3451	1	0.11197	1.0087	0.3858
**Rhizosphere**								
G1 drought	1	0.16482	1.5787	**0.021**	1	0.13206	1.2172	0.1011

Table S11. Effects of drought treatment (G1 drought = parent drought treatment, G2 drought = current drought treatment), plant genotype, and their interactions on beta diversity (Bray-Curtis distance matrix) of generation 2 (G2) plant were assessed by PERMANOVA (df = degrees of freedom, R^2 = coefficient of determination, F = F-statistic, P = P-value, number of permutations = 9999, values P<0.05 are indicated in bold).

PERMANOVA	Pays de la Loire (France)				PERMANOVA	Michigan (USA)			
	df	R^2	F	P		df	R^2	F	P
**Root**					**Root**				
Genotype	1	0.30600	15.4685	**0.0006**	*G1 drought*	1	0.01702	0.7980	0.2832
G1 drought	1	0.01027	0.5190	0.5053	*G2 drought*	1	0.02670	1.2517	**0.0290**
G2 drought	1	0.00607	0.3067	0.6443	*G1 drought x G2 drought*	1	0.01767	0.8283	0.2239
Genotype x G1 drought	1	0.01047	0.5292	0.4970					
Genotype x G2 drought	1	0.02327	1.1762	0.2767					
G1 drought x G2 drought	1	0.12623	6.3810	**0.0111**					
Genotype x G1 drought x G2 drought	1	0.00337	0.1704	0.8049					
**Rhizosphere**					**Rhizosphere**				
Genotype	1	0.06125	2.5661	**0.0007**	*G1 drought*	1	0.01450	0.6848	0.5682
G1 drought	1	0.06888	2.8856	**0.0002**	*G2 drought*	1	0.01542	0.7281	0.3975
G2 drought	1	0.05483	2.2972	**0.0031**	*G1 drought x G2 drought*	1	0.01708	0.8065	0.1801
Genotype x G1 drought	1	0.06560	2.7483	**0.0006**					
Genotype x G2 drought	1	0.01959	0.8206	0.6887					
G1 drought x G2 drought	1	0.02926	1.2259	0.2074					
Genotype x G1 drought x G2 drought	1	0.03224	1.3505	0.1358					

Table S12. Effects of G1 drought (parent drought treatment), G2 drought (current drought treatment), and their interactions on beta diversity (Bray-Curtis distance matrix) of generation 2 (G2) plant within each genotype in Pays de la Loire, France were assessed by PERMANOVA (df = degrees of freedom, R^2 = coefficient of determination, F = F-statistic, P = P-value, number of permutations = 9999, values P<0.05 are indicated in bold).

PERMANOVA	Pays de la Loire (France)							
	Flavert				Red Hawk			
	df	R^2	F	P	df	R^2	F	P
**Root**								
G1 drought	1	0.03099	0.6254	0.4759	1	0.02719	0.4127	0.5974
G2 drought	1	0.03024	0.6102	0.4803	1	0.05641	0.8560	0.3836
G1 drought x G2 drought	1	0.24490	4.9412	**0.0272**	1	0.12569	1.9075	0.1750
**Rhizosphere**								
G1 drought	1	0.20094	4.3202	**0.0001**	1	0.05668	0.9956	0.4454
G2 drought	1	0.07582	1.6302	**0.0497**	1	0.08992	1.5795	0.0525
G1 drought x G2 drought	1	0.07205	1.5491	0.0536	1	0.05635	0.9898	0.4634

Table S13. Beta dispersion analysis of generation 1 (G1) plants were assessed by PERMDISP (df = degrees of freedom, F = F-statistic, P = P-value, number of permutations = 999, values P<0.05 are indicated in bold)

PERMADISP	Pays de la Loire (France)						PERMADISP	Michigan (USA)		
	Flavert			Red Hawk				Red Hawk		
	df	F	P	df	F	P		df	F	P
**Root**										
G1 drought	1	0.798	0.448	1	0.6708	0.494	*G1 drought*	1	0.5402	0.488
**Rhizosphere**										
G1 drought	1	4.0062	**0.046**	1	1.3403	0.301	*G1 drought*	1	0.0581	0.817

Table S14. Beta dispersion analysis of generation 2 (G2) plants were assessed by PERMDISP (df = degrees of freedom, F = F-statistic, P = P-value, number of permutations = 999, values P<0.05 are indicated in bold)

**PERMADISP**	**Pays de la Loire (France)**						**PERMADISP**	**Michigan (USA)**		
	**Flavert**			**Red Hawk**				**Red Hawk**		
	**df**	**F**	**P**	**df**	**F**	**P**		**df**	**F**	**P**
**Root**							**Root**			
G1 drought	1	0.0886	0.759	1	0.5329	0.478	*G1 drought*	1	1.1104	0.292
G2 drought	1	0.0091	0.941	1	1.8366	0.198	*G2 drought*	1	1.0537	0.309
G1 X G2 drought	3	0.8629	0.496	3	1.6994	0.211	*G1 X G2 drought*	3	1.2683	0.265
**Rhizosphere**							**Rhizosphere**			
G1 drought	1	0.5645	0.488	1	0.0022	0.96	*G1 drought*	1	2.6643	0.113
G2 drought	1	4.0549	**0.048**	1	1.8265	0.181	*G2 drought*	1	5.1359	**0.028**
G1 X G2 drought	3	4.1156	**0.02**	3	0.2518	0.843	*G1 X G2 drought*	3	1.6794	0.179
